# Selected Gut Bacteria from Water Monitor Lizard Exhibit Effects against Pathogenic *Acanthamoeba castellanii* Belonging to the T4 Genotype

**DOI:** 10.3390/microorganisms11041072

**Published:** 2023-04-20

**Authors:** Noor Akbar, Naveed Ahmed Khan, Alexander D. Giddey, Nelson C. Soares, Ahmad M. Alharbi, Hasan Alfahemi, Ruqaiyyah Siddiqui

**Affiliations:** 1 Department of Clinical Sciences, College of Medicine, University of Sharjah, University City, Sharjah 27272, United Arab Emirates; 2 Sharjah Institute for Medical Research, University of Sharjah, Sharjah 27272, United Arab Emirates; 3 Department of Medical Biology, Faculty of Medicine, Istinye University, Istanbul 34010, Turkey; 4 Department of Medicinal Chemistry, College of Pharmacy, University of Sharjah, Sharjah 27272, United Arab Emirates; 5 Department of Clinical Laboratory Sciences, College of Applied Medical Sciences, Taif University, Taif 21944, Saudi Arabia; 6 Department of Medical Microbiology, Faculty of Medicine, Al-Baha University, P.O. Box. 1988, Al-Baha 65799, Saudi Arabia; 7 College of Arts and Sciences, American University of Sharjah, University City, Sharjah 26666, United Arab Emirates

**Keywords:** gut microbiota, *Acanthamoeba castellanii*, water monitor lizard, cytopathogenicity

## Abstract

Water monitor lizards (WMLs) reside in unhygienic and challenging ecological surroundings and are routinely exposed to various pathogenic microorganisms. It is possible that their gut microbiota produces substances to counter microbial infections. Here we determine whether selected gut bacteria of water monitor lizards (WMLs) possess anti-amoebic properties using *Acanthamoeba castellanii* of the T4 genotype. Conditioned media (CM) were prepared from bacteria isolated from WML. The CM were tested using amoebicidal, adhesion, encystation, excystation, cell cytotoxicity and amoeba-mediated host cell cytotoxicity assays *in vitro*. Amoebicidal assays revealed that CM exhibited anti-amoebic effects. CM inhibited both excystation and encystation in *A. castellanii*. CM inhibited amoebae binding to and cytotoxicity of host cells. In contrast, CM alone showed limited toxic effects against human cells *in vitro*. Mass spectrometry revealed several antimicrobials, anticancer, neurotransmitters, anti-depressant and other metabolites with biological functions. Overall, these findings imply that bacteria from unusual places, such as WML gut, produce molecules with anti-acanthamoebic capabilities.

## 1. Introduction

*Acanthamoeba* is pervasive in a variety of environments [1,2,3]. Its life cycle includes a dormant cyst stage and an active trophozoite stage [1,4]. Pathogenic *Acanthamoeba* can cause granulomatous amoebic encephalitis (GAE) and blinding keratitis [1]. Despite advances in antimicrobial chemotherapy, treatment remains problematic, suggesting the need to find therapeutic agents [3,5,6].

Secondary metabolites are produced by a variety of organisms, including different animals, microorganisms (bacteria, fungi and parasites, etc.), marine organisms, terrestrial plants, vertebrate and invertebrate species [7,8,9,10,11,12]. Although plant-derived products have been used frequently in folk medicine, the discovery of penicillin signaled a significant shift in the source of natural molecules from plants to microorganisms [13]. Since then, metabolites produced by bacteria have been widely used in the food industries, agriculture, pharmaceuticals and scientific research [14]. For example, *Bacillus*, cyanobacteria, myxobacteria and *Pseudomonas* produce secondary metabolites with therapeutic values [15]. *Streptomyces venezuelae* produced pikromycin, the first known polyketide antibiotic [16]. *Streptomyces noursei* produced a potent polyene antifungal agent, i.e., Nystatin [17]. Amphotericin B is another antifungal drug produced by *Streptomyces nodosus* [18]. *Pseudomonas* species isolated from the Antarctic region produced broad-spectrum antiparasitic and antiproliferative effects [19]. *Streptomyces hachijoensis* derived Trichomycin exhibited antifungal and antitrichomonal activity [20]. 

Water monitor lizards (WMLs) breathe in unhygienic and challenging ecological surroundings, commonly feeding on rotten food contaminated with various pathogenic microorganisms. Therefore, the purpose of this investigation was to extract and characterize the selected gut bacterial species of WML. CM were tested against pathogenic *A. castellanii* of the genotype T4 in different assays to determine their anti-amoebic potential.

## 2. Materials and Methods

### 2.1. Dissection of Water Monitor Lizard and Isolation of Gut Bacteria

In brief, the Department of Wildlife and National Parks (PERHILITAN), Malaysia, approved this study, as well as Sunway University, Malaysia (SUNREC 2019/023). Prior to the experiments, all operating and dissecting tools were properly sterilized in an autoclave. Additionally, during the dissection procedure, tools were surface sterilized with 70% ethanol. WML was dissected, gut was removed using the aseptic technique and culturable bacteria were isolated with sterile swabs. The swabs were cultured on blood agar plates, and the plates were incubated at 37 °C for 24 h. Bacterial colonies were differentiated from each other, and pure cultures were grown in nutrient broth overnight at 37 °C.

### 2.2. DNA Extraction, Amplification and Identification of Isolated Bacteria

DNA were extracted from pure bacterial cultures using the AllPrep Bacterial DNA extraction kit (Qiagen, Germantown, MD, USA). The extracted DNA were amplified using 16S rRNA universal primers (27F 5′ AGAGTTTGATCMTGGCTCAG 3′ and 1492R 5′ TACGGYTACCTTGTTACGACTT 3′) [21]. The amplified products were sequenced commercially (1st Base; Axil Scientific Pt. Ltd., Singapore). In order to find matches with pre-existing reference sequences, the output sequences were exported into the alignment program Basic Local Alignment Search Tool (BLAST). Using the Maximum Likelihood (ML) approach in MEGA 7 and the GTR+G model with concatenated 16S rRNA sequences, the phylogenetic tree was reconstructed.

### 2.3. Preparation of Bacterial Conditioned Media

Pure bacterial colonies were cultured in Roswell Park Memorial Institute (RPMI) at 37 °C with constant shaking at 90 rpm for 48 h [11,12,22]. Next, bacterial cultures were centrifuged at 10,000× *g* for 60 min at 4 °C. Cell free culture supernatants were collected and filtered using 0.22 μm pore size filters and kept at −80 °C until tested.

### 2.4. Acanthamoeba castellanii Cultures

*A. castellanii* genotype T4 (ATCC 50492) was grown in a proteose-peptone-yeast-glucose (PYG) growth medium containing 0.75% yeast extract, 1.5% d-glucose and 0.75% proteose-peptone [6]. Cultures were kept at 30 °C until confluent. For assays, adhering trophozoites were detached by placing the flask on ice for 10 min and then gentle tapping. The culture containing amoebae was then centrifuged at 2500× *g* for 5 min. Next, supernatants were discarded, and the pellet was re-suspended in 1 mL of RPMI medium and amoebae were enumerated using a hemocytometer.

### 2.5. Amoebicidal Assays

Amoebicidal assays were performed as described previously [6,22]. Briefly, 5 × 10^5^ amoebae were incubated with different CM for 2 and 24 h at 30 °C. Controls include amoebae alone in RPMI (negative control) and with 25 µM chlorhexidine (positive control). Finally, 0.1% Trypan blue dye was added to each well, and amoebae were enumerated using a hemocytometer. The number of viable unstained amoebae was determined as before [6].

### 2.6. HeLa Cell Cultivation

HeLa cells were obtained from the American Type Culture Collection (ATCC CCL-2) and maintained in RPMI with L-glutamine complemented with 1% minimum essential amino acids, 10% fetal bovine serum (FBS) and 1% Penicillin-Streptomycin (Pen-Strep) at 37 °C in an incubator with 5% CO_2_ [6].

### 2.7. Adhesion Assays

CM were tested in adhesion assays to see their effects on amoebae binding to human cells [23]. Briefly, 100 µL of each CM was incubated with 5 × 10^5^ amoebae at 30 °C for 120 min. Next, amoebae were centrifuged at 2500× *g* for 5 min, and pelleted amoebae (pre-treated amoebae) were obtained. The pellet was resuspended in RPMI (200 µL) and subsequently transferred to HeLa cells. Plates were incubated at 37 °C for 60 min. Finally, unbound amoebae were enumerated using a hemocytometer, and the percentage amoebae was estimated using the given equation: % amoebae (bound) = 100 − amoebae (unbound).

### 2.8. Encystation Assays

Using 16% glucose as an encystation medium, approximately 1 × 10^6^ amoeba trophozoites were incubated with different CM for 48 h at 30 °C as before [24]. Next, amoebae were treated with sodium dodecyl sulphate (SDS) (0.1%) for approximately 20 min. Using a hemocytometer, the residual cysts were counted, and the results were recorded.

### 2.9. Excystation Assays

To prepare cysts, *A. castellanii* trophozoites were placed on non-nutrient agar plates for two weeks at 30 °C, as described earlier [5,6]. Cysts were collected by scrapping off the agar plates using a cell scrapper using PBS. Cysts were pelleted by centrifugation at 3000× *g* for 10 min. Next, half a million cysts were incubated with 100 µL of different CM in PYG medium with a final volume of 500 µL for up to 72 h [6]. Finally, a hemocytometer was used to count viable amoebae trophozoites.

### 2.10. Host Cell Cytotoxicity Assays

To determine the cytotoxic effects of CM, 100 µL of the CM were incubated with HeLa cell monolayers in 96-well plate as described earlier [6,11]. The plate was maintained for an overnight period in the cell culture incubator supplied with 5% CO_2_ at 37 °C. Finally, cell supernatants were collected, and LDH release was determined using Cytotoxicity Detection kit (Roche Diagnostics, Indianapolis, IN, USA). Percent cell damage was determined by the following equation,

(1) $$\%\;Cytotoxicity=\frac{sample\;mean\;value\;-\;negative\;control\;mean\;value}{positive\;control\;mean\;value\;-\;negative\;control\;mean\;value}\times 100$$

### 2.11. Cytopathogenicity Assays

Cytopathogenicity assays were performed to determine whether CM inhibits amoebae-mediated host cell death. In brief, amoebae were treated with CM as described in adhesion assays, and then, pre-treated amoebae were incubated with HeLa cell monolayers overnight at 37 °C. The amoebae-mediated cell damage was evaluated by measuring the amount of LDH enzyme released into cell media by damaged cells as before ([25]).

### 2.12. Ultra-Performance Liquid Chromatography Tandem Mass Spectrometry (HPLC-MS/MS)

For metabolomics, the sample metabolite extraction was carried out as before [26]. Next, each sample was given two injections of 1 µL for metabolomics, which were followed by 30 min of elution using the gradient described below: Over the course of 15 min, ACN was raised from 1% to 99%, held there for 3 min, and then reduced to 1% ACN for another 10 min. Elution and re-equilibration flow rates were 250 and 350 µL/min, respectively. The analysis was carried out using an Apollo II electrospray ionization (ESI) source and a TimsTOF (Bruker, Darmstadt, Germany). The drying temperature, gas flow and nebulizer pressure levels were adjusted to 220 °C, 10 µL/min and 2.2 bar, respectively. The capillary voltage and end plate offset were 4500 V and 500 V, respectively. For metabolites processing and statistical assessment, MetaboScape^®^ 4.0 was utilized. The T-ReX 2D/3D method for molecular characterization and “bucketing” was modified using the following criteria: Peak identification requires a minimum peak length of 7 spectra and a minimum intensity threshold of 1000 counts with characteristic verification based on peak area. The file masses were recalibrated using an external calibrant that was applied between 0 and 0.3 min. Only data having retention times somewhere between 0.3 and 25 min and *m*/*z* ranges between 50 and 1000 were properly considered, whereas the MS/MS import strategy was set to average. All the experimentations were performed in three biological replicates while each sample (extracts) was analyzed in duplicate using LC-TIMS-QTOF MS.

MetaboScape compared features to the MetaboPharmHam database (HMDB) using retention time and accurate mass or MS2 spectral identification. Then, among the numerous other entries within the same molecule, the data of the same compounds that perfectly matched the maximum number of variables, such as *m*/*z* values, MS/MS, analyte list, spectrum library and retention time were chosen as the metabolite entries to be further filtered. T-test (two sample, two-tailed distribution) statistical analyses were performed to highlight the impact of CM on amoebae. The data are shown as the average standard deviation of multiple replicated experiments. The software Graph Pad Prism version 8.0 was used for all of the analyses and visualizations. *p* ≤ 0.05 was chosen as the statistical significance threshold.

## 3. Results

### 3.1. Isolation and Identifcation of Bacterial Isolates Derived from the Gut of Water Monitor Lizard

**Figure 1:**
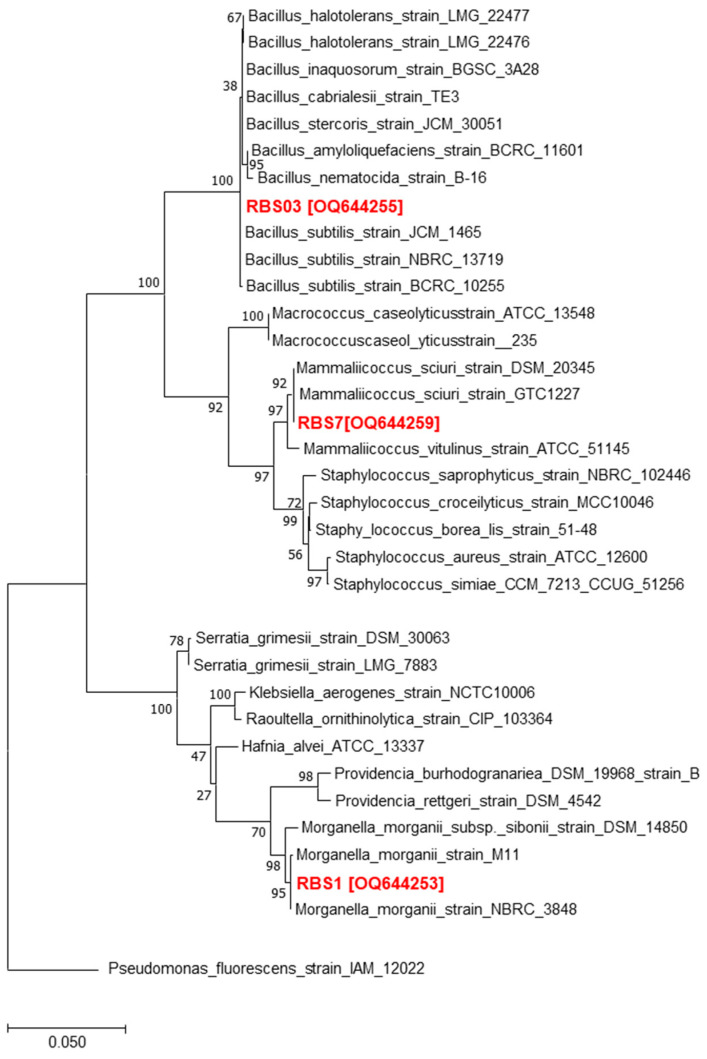
Based on the phylogenetic analysis of their 16 S rRNA genes, the phylogenetic tree shows the isolated bacteria from the water monitor lizard stomach that were employed in this work (red) and Pseudomonas fluorescens as an outgroup. The GTR+G model with concatenated 16 S rRNA sequences was used as the foundation for the dendrogram reconstruction using the Maximum Likelihood (ML) approach (MEGA 7). Branching nodes show percentage bootstrap values higher than 50% of 1000 repetitions.

Several bacterial species were isolated from water monitor lizard gut. Sequencing results revealed numerous bacterial species (Table 1). Several bacteria, including *Morganella morganii, Bacillus subtilis* and *Mammaliicoccus sciuri*, were identified (Figure 1 and Table 1).

### 3.2. Conditioned Media Isolated from Selected Gut Bacteria of Water Monitor Lizard Showed Amoebicidal Activity

**Figure 2:**
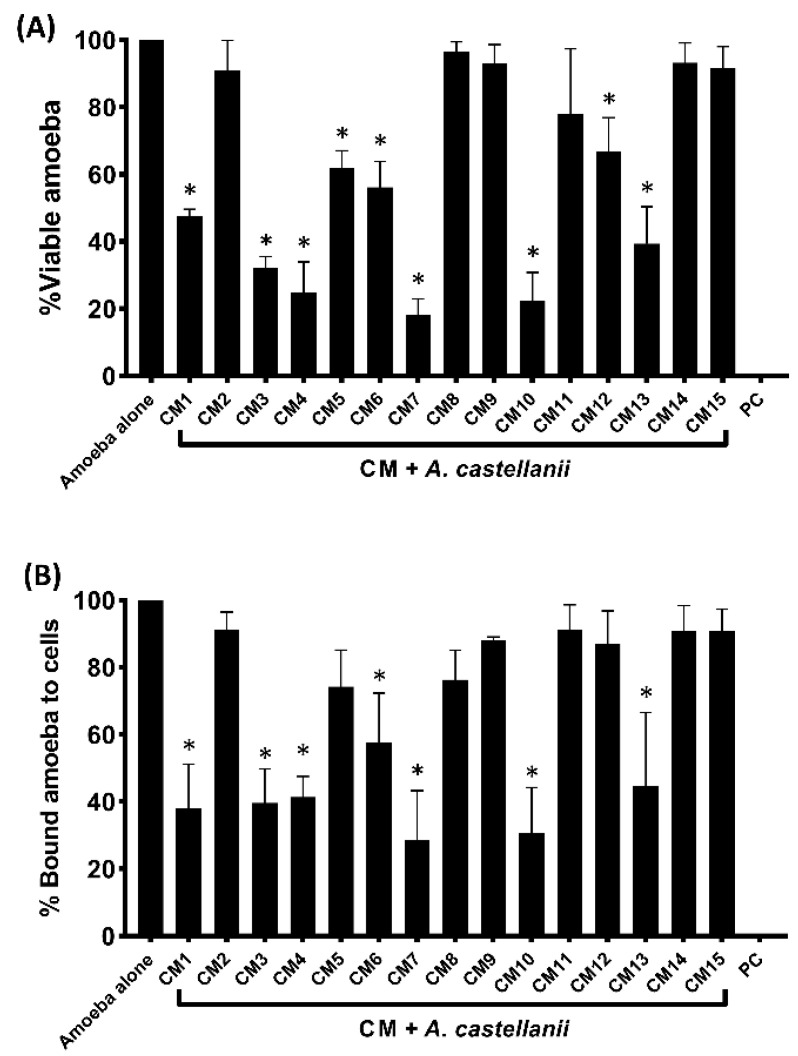
**(A**) Conditioned media were prepared by incubating each bacterium in RPMI at 37 °C for 48 h. Next, bacterial cultures were centrifuged for 60 min at 10,000× *g* at 4 °C, and supernatants, i.e., CM, were filtered using 0.22 μm pore size filters. Equal doses (100 µL) of various bacterial CM were used to determine amoebic effects. Briefly, *A. castellanii*, i.e., 5 × 10^5^, were incubated with 100 µL of CM for 24 h at 30 °C. After this, viable amoebae were counted using a hemocytometer microscopically. Amoebae grown in RPMI alone were taken as the negative control, while amoebae incubated with 25 µM chlorhexidine were used as the positive control. The data are expressed as the mean ± standard error. *p* values were determined using two-sample *t*-test, two-tailed distribution, and (*) is *p* ≤ 0.05. *p* ≤ 0.05 was chosen as the statistical significance threshold. (**B**) Conditioned media from water monitor lizard gut bacteria repressed the *A. castellanii* binding to human cells. Adhesion assays were performed to determine whether *A. castellanii* interacts with human cells. Note that CM repressed *A. castellanii* significantly. *p* values were determined using two-sample *t*-test, two-tailed distribution, (*) is <0.05. The data are expressed as the mean ± standard error.

The findings showed that against *A. castellanii*, CM1, CM3-CM7, CM10, CM12 and CM13 showed anti-amoebic effects (Figure 2A). CM7 reduced amoeba viability by 82%, followed by CM10 (78%), CM4 (76%), CM3 (68%) and CM1 (53%). Contrarily, CM5, CM6, and CM12 demonstrated 38%, 44%, and 33% amoebicidal effects (Figure 2A).

### 3.3. Bacterial Conditioned Media Inhibited the Binding of Amoebae to Human Cells

The findings showed that with the exception of CM5 and CM12, all CM with amoebicidal activity decreased amoebae adhesion to HeLa cells (Figure 2B). CM7 and CM10 reduced amoeba binding by 72% and 69%, respectively, while CM1, CM3, CM4, CM13 and CM6 inhibited amoebae adhesion to human cells by 62%, 61%, 59%, 56% and 42%, respectively (Figure 2B). Amoeba attachment to HeLa cells was not prevented by the remaining CM (Figure 2B).

### 3.4. Encystation and Excystation of Amoebae were Inhibited

**Figure 3:**
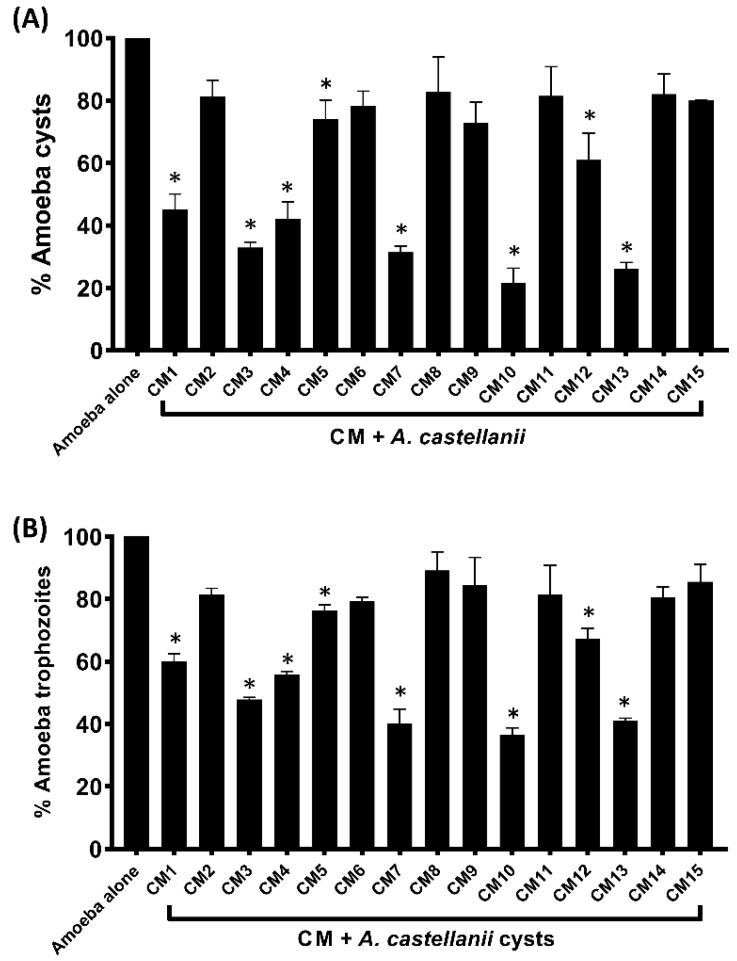
(**A**) Effects of CM on the encystation of *A. castellanii* trophozoites. The results showed that CM inhibited the encystment of trophozoite form compared to the negative control. The data are presented as the mean ± standard error. *p* values were calculated using a two-sample *t*-test with a two-tailed distribution, and (*) denotes that *p* ≤ 0.05. (**B**) Effects of CM on *A. castellanii* excystation. When compared to the negative control, the results demonstrated that CM reduced the excystment of trophozoites. Overall, the CM prevented the excystation process in amoebae. The data are presented as the mean ± standard error. *p* values were calculated using a two-sample *t*-test with a two-tailed distribution, and (*) denotes that *p* ≤ 0.05.

*A. castellanii* trophozoites treated with 16% glucose (encystation media) were used as the negative control in encystation experiments, and *A. castellanii* cysts obtained were considered to be 100%. The results revealed that several CM inhibited *A. castellanii* encystation when compared to the negative control (Figure 3A). In the presence of CM10 and CM13, cysts formation declined to 21% and 26%, respectively. Similarly, CM1, CM3, CM4, CM7 and CM12 arrested 55%, 67%, 58%, 69% and 39% amoebae (Figure 3B).

### 3.5. Conditioned Media Exhibited Limited Cytotoxicity and Inhibited Amoebae–Mediated Host Cell Damage

**Figure 4:**
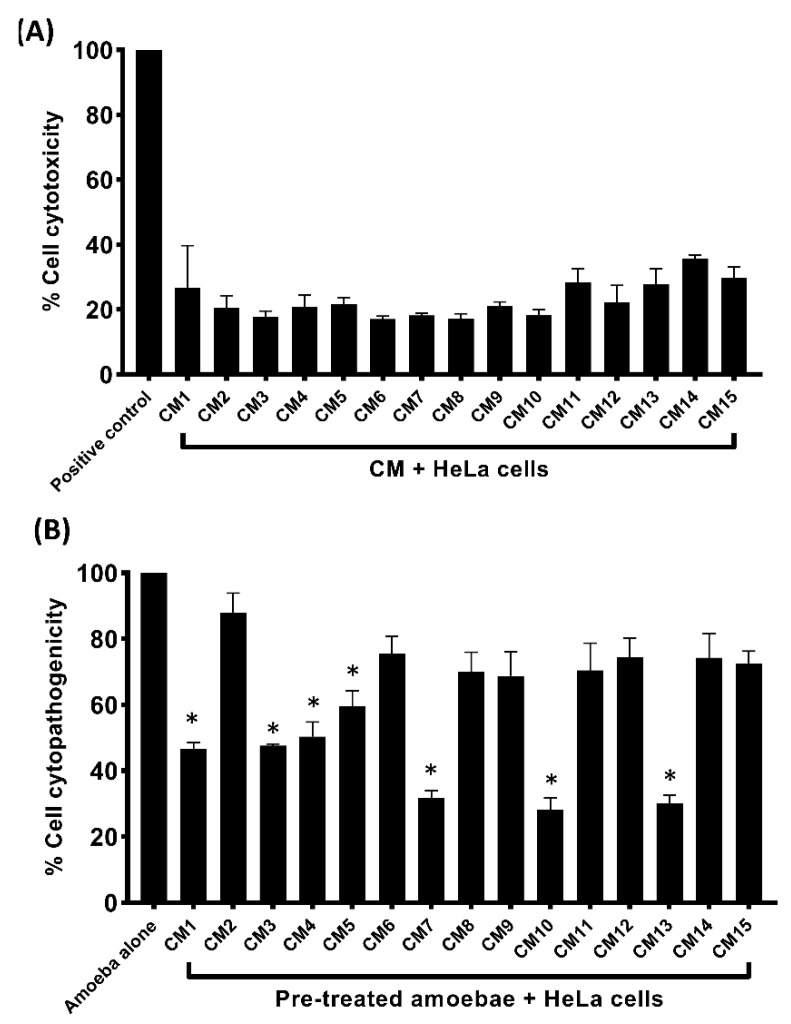
(**A**) The effects of CM on human cell lines. The compounds alone and in complex form exhibited limited cytotoxicity. (**B**) The cytopathogenicity of human cells was decreased by pre-treating *A. castellanii* with CM. The results revealed substantial inhibition of amoeba-mediated host cytotoxicity when compared to amoeba (untreated). Data are presented as mean  ±  standard error. (*) denotes where *p* ≤ 0.05.

The CM produced minimal toxicity against HeLa cell lines, except CM1, CM11 and CM13-15, which exhibited low to moderate cell cytotoxicity (Figure 4A). Similarly, CM decreased host cell cytotoxicity caused by amoebae (Figure 4B). Less than 30% of the cells in CM7, CM10 and CM13 were found to have damage compared to CM1 and CM3, which limited the damage to 46% (Figure 4B).

### 3.6. Mass Spectrometry Revealed Numerous Metabolites

Mass spectrometry revealed a total of 189 tentatively identified metabolites (Table 2 and Supplementary Table S1). Among the identified metabolites, the most abundant and important compounds were Carvone, 1,11-Undecanedicarboxylic acid, 4-Ethylphenol, Benzoic acid, Hydrocinnamic acid, Indole-3-carbinol, Indolelactic acid, Quinaldic acid, Succinic acid and several other compounds, some of which with known antimicrobial effects (Table 2 and Supplementary Table S1). Principal Components Analysis (PCA) shows the relative (dis)similarity between samples according to the first two principal components. Points represent samples coloured according to the location of the point of origin. Circles represent Gram-negative and triangles Gram-positive bacterial species (Supplementary Figure S1a). Among these metabolites, there are 12 metabolites with significant altered abundance across sampling locations. Androsterone is more abundant in samples taken from the mouth and oesophagus while nearly absent in samples from other locations. Estrone is nearly absent in samples drawn from the anus/rectum and large intestine but abundant in samples from all other locations (Supplementary Figure S1b). Furthermore, hierarchical clustering shows that bacteria collected from the mouth generally clustered together, as did samples from the anus/rectum and large intestine, with samples from origins between those extremes mostly forming a cluster between those two (Supplementary Figure S2).

## 4. Discussion

*Acanthamoeba*, being a free-living amoeba, can be found in a wide range of habitats, including water, soil, swimming pools and hospitals [47]. It can endure extreme conditions and cause eye and brain infections [4]. Many available drugs are used to fight amoebic infections, but their non-specificity and potent cytotoxicity are challenges to researchers [5]. As a result, new anti-amoebic drugs with high bioactivity are required. In the present study, conditioned media from WML gut bacteria were prepared and tested against the pathogenic *A. castellanii* genotype T4. The results have shown that CM from water monitor gut bacteria showed remarkable amoebicidal effects against *A. castellanii*. CM also restricted the binding capability of the parasite to human cell lines and amoeba-mediated host cell death. Furthermore, the CM inhibited both the encystation and excystation processes in amoebae and showed limited cytotoxicity towards HeLa cells. These findings are consistent with earlier research [11,22,48]. CM isolated from bacteria identified from WML intestine indicated considerable anticancer effects [49]. *Streptomyces avermitilis* produces avermectins and their derivatives that possess strong anti-parasitic activity against *Onchocerca volvulus* (parasite) [50]. *Saccharopolyspora spinose* produces spinosad that exhibited notable insecticidal activity against ecto-parasite of the livestock [14].

In this study, CM produced by the gut microbiota of WML showed significant amoebicidal action against pathogenic *A. castellanii*. In our previous study, CM from cockroach gut bacteria possessed important amoebicidal activity against *A. castellanii* [22]. Iqbal et al. reported that the CM from *E. coli* K1 and *Enterobacter* species exhibited noteworthy amoebicidal and amoebistatic effects against *A. castellanii* [51]. Therefore, our findings are consistent with earlier findings emphasizing the significance of WML gut bacteria as an anti-amoebic agent(s).

Also, we investigated the effects of the CM isolated from WML gut bacteria on the adhesion capabilities of *A. castellanii* to human cells. Following therapy, CM precluded amoebae from attaching to human cells, reducing cell damage. Matin et al. reported the interaction of human brain microvascular endothelial cells (HBMECs) and *Balamuthia mandrillaris*, where galactose impeded the binding capability of *B. mandrillaris* to HBMECs [52].

Conditioned medium minimized amoeba-mediated host cell damage and inhibited the excystation and encystation of *A. castellanii* while exhibiting low cytotoxicity. Quercetin and kolavenic acid, which were extracted from *Polyalthia longifolia*, have virtually no harmful effects on human cell lines [24]. Similarly, CM of bacteria isolated from animals gut produced minimal cytotoxic properties against human cells [11,12,21,48].

It is interesting to note that the isolated bacteria from WML gut produced a variety of bioactive metabolites with antimicrobial effects. These metabolites include benzoic acid [33,34,35,36,37,39], carvone [27], 4-Ethylphenol [32], succinic acid [46] and 1,11-Undecanedicarboxylic acid etc. [29,30]. These bacteria also produced anticancer molecules, such as Carvone and Indole-3-carbinol [27,41,42], enzyme inhibitors [38] and various pharmacologically and commercially important metabolites. The compound 4-Ethylphenol produced by *Lactobacillus plantarum* is a strong odorant and is used as a strong anti-fungal agent [31,32]. Likewise, *Candida* species produced Indolelactic acid, which exhibited antibacterial effects against Gram-positive and Gram-negative bacteria [44]. Quinaldic acid extracted from *Ephedra pachyclada* showed notable antibacterial activity against intestinal bacteria [45]. Likewise, various antimicrobial (antibacterial and antifungal) lipopeptides (surfactin and iturin A) were generated by *Bacillus subtills* found in *Rattus rattus* (rat) intestine [21].

The identified molecules revealed different modes of action. For example, succinic acid identified from dried and pickled mustard damaged the cell membrane in *Staphylococcus aureus* and *Pseudomonas fluorescens* [52]. Similarly, Indole-3-carbinol showed anti-cancer activity by arresting the cells at G1 phase and inhibiting the expression of cyclin-dependent kinase-6 [53]. Benzoic acid showed antimicrobial effects against *Zygosaccharomyces bailii* by depleting ATP generation [54]. In this study, these metabolites have been identified from WML gut bacteria. The anti-amoebic effects may be due to the presence of such bioactive compounds that could lead to membrane damage, depletion of energy and inhibition of growth related enzymes or other targets. Several metabolites with antimicrobial, anticancer and other well-known biological activities were identified. Our findings propose that microbial species from unexplored sources, such as the gut of a water monitor lizard, produce anti-amoebic compounds that have the potential to become potent anti-parasitic medicines.

## Figures and Tables

**Table 1:** Bacterial species isolated from the gut of water monitor lizard.

S. No.	Conditioned Media Code	Bacteria Species	Accession Number
1.	CM1	*Morganella morganii*	OQ644253
2.	CM2	*Empedobacter brevis*	OQ644254
3.	CM3	*Bacillus subtilis*	OQ644255
4.	CM4	*Pseudomonas otitidis*	OQ644256
5.	CM5	*Pseudoclavibacter alba*	OQ644257
6.	CM6	*Pseudomonas aeruginosa*	OQ644258
7.	CM7	*Mammaliicoccus sciuri*	OQ644259
8.	CM8	*Proteus terrae*	OQ644260
9.	CM9	*Chryseobacterium taklimakanense*	OQ644261
10.	CM10	*Bacillus aerius*	OQ644262
11.	CM11	*Morganella morganii*	OQ644263
12.	CM12	*Corynebacterium atrinae*	OQ644264
13.	CM13	*Mammaliicoccus sciuri*	OQ644265
14.	CM14	*Chryseobacterium taklimakanense*	OQ644266
15.	CM15	*Corynebacterium freneyi*	OQ644267

**Table 2:** Mass spectrometry revealed several compounds with biological activities.

Compounds Names	RT [min]	*m*/*z* Meas.	Chemical Formula	Structure	Activity
(+)-(S)-Carvone	11.9	151.113	C_10_H_14_O	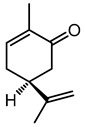	Antimicrobial activity (Antibacterial, antifungal, antiparasitic) by damaging plasma membrane [27], neurological activity [28], anti-neuraminidase, antioxidant, anti-inflammatory and anticancer activities [27].
1,11-Undecanedicarboxylic acid	10.47	245.175	C_13_H_24_O_4_	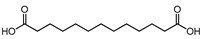	The analogues exhibited antimicrobial activity [29,30].
4-Ethylphenol	9.88	123.0804	C_8_H_10_O	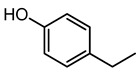	This is a strong odorant compound produced by *Lactobacillus plantarum* [31]. This is an agrochemical agent produced by the disease-resistant soybean eliminating fungal infections caused by *P. sojae* and *Phytophthora nicotianae.* The volatile compound has potent anti-fungal effects against soil-borne fungi, i.e., *Rhizoctonia solani, Gaeumannomyces graminis var tritici* and *Fusarium graminearum* [32].
Benzoic acid	3.41	123.0484	C_7_H_6_O_2_	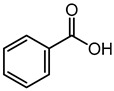	Benzoic acid alone is considered to be a general-purpose antimicrobial agent with a broad spectrum of actions towards human pathogenic bacteria and fungi with varying minimum inhibitory concentrations (MIC) values [33,34,35,36,37]. Additionally, it was being tested as a potential inhibitor of -carbonic anhydrase, a novel molecular target found in Cryptococcus neoformans and C. albicans [38]. 2,4-dihidroxy benzoic acid showed significant antibacterial activity against *Vibrio alginolyticus* and *E. coli* [39].
Hydrocinnamic acid	5.95	151.0728	C_9_H_10_O_2_	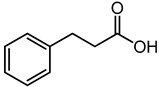	It belongs to the phenylpropanoids class, having an important role in pharmaceuticals, cosmetics and as a food additive [40].
Indole-3-carbinol	9.52	148.076	C_9_H_9_NO	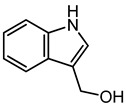	It is used to inhibit several types of cancers in human and prevent carcinogenesis in animal models [41,42]. The dimeric counterpart of indole-3-carbinol, i.e., 3,3′-diindolylmethane, is a very crucial anti-cancer agent [43].
Indolelactic acid	7.31	206.084	C_11_H_11_NO_3_	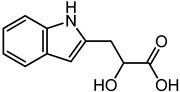	*Candida* species produce Indolelactic acid that inhibit several Gram-negative and Gram-positive bacteria [44].
Quinaldic acid	5.98	174.0551	C_10_H_7_NO_2_	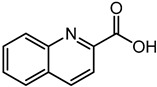	The Quinaldic acid inhibit the growth of several intestinal bacteria [45].
Succinic acid	8.36	119.0351	C_4_H_6_O_4_	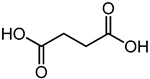	Succinic acid has a strong antibacterial effect against *E. coli* [46].

## Data Availability

The data described in this study are accessible from the corresponding author upon request.

## Supplementary Materials

The following supporting information can be downloaded at: https://www.mdpi.com/article/10.3390/microorganisms11041072/s1.
