# Supplementary material for: Selected Gut Bacteria from Water Monitor Lizard Exhibit Effects against Pathogenic Acanthamoeba castellanii Belonging to the T4 Genotype
Source: Microorganisms. 2023 Apr 20;11(4):1072. doi: 10.3390/microorganisms11041072 (PMC10142573; doi:10.3390/microorganisms11041072)
Supplement: Supplementary file 1 [file microorganisms-11-01072-s001.zip › Supplementary Fig S1.pptx]

## Slide 1
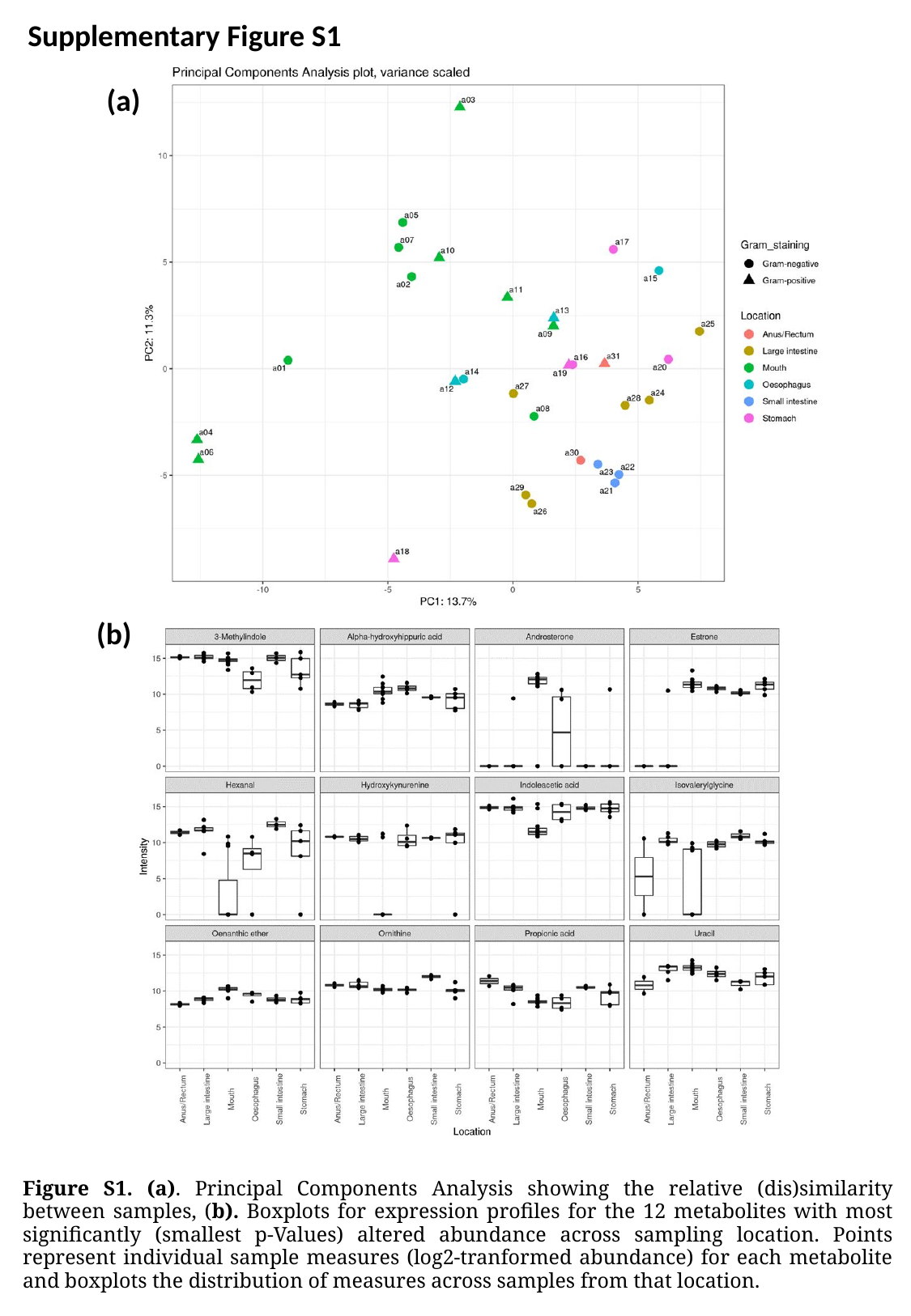

Supplementary Figure S1
(a)
(b)
Figure S1. (a). Principal Components Analysis showing the relative (dis)similarity between samples, (b). Boxplots for expression profiles for the 12 metabolites with most significantly (smallest p-Values) altered abundance across sampling location. Points represent individual sample measures (log2-tranformed abundance) for each metabolite and boxplots the distribution of measures across samples from that location.
