# Supplementary material for: Selected Gut Bacteria from Water Monitor Lizard Exhibit Effects against Pathogenic Acanthamoeba castellanii Belonging to the T4 Genotype
Source: Microorganisms. 2023 Apr 20;11(4):1072. doi: 10.3390/microorganisms11041072 (PMC10142573; doi:10.3390/microorganisms11041072)
Supplement: Supplementary file 1 [file microorganisms-11-01072-s001.zip › Supplementary Fig S2.pptx]

## Slide 1
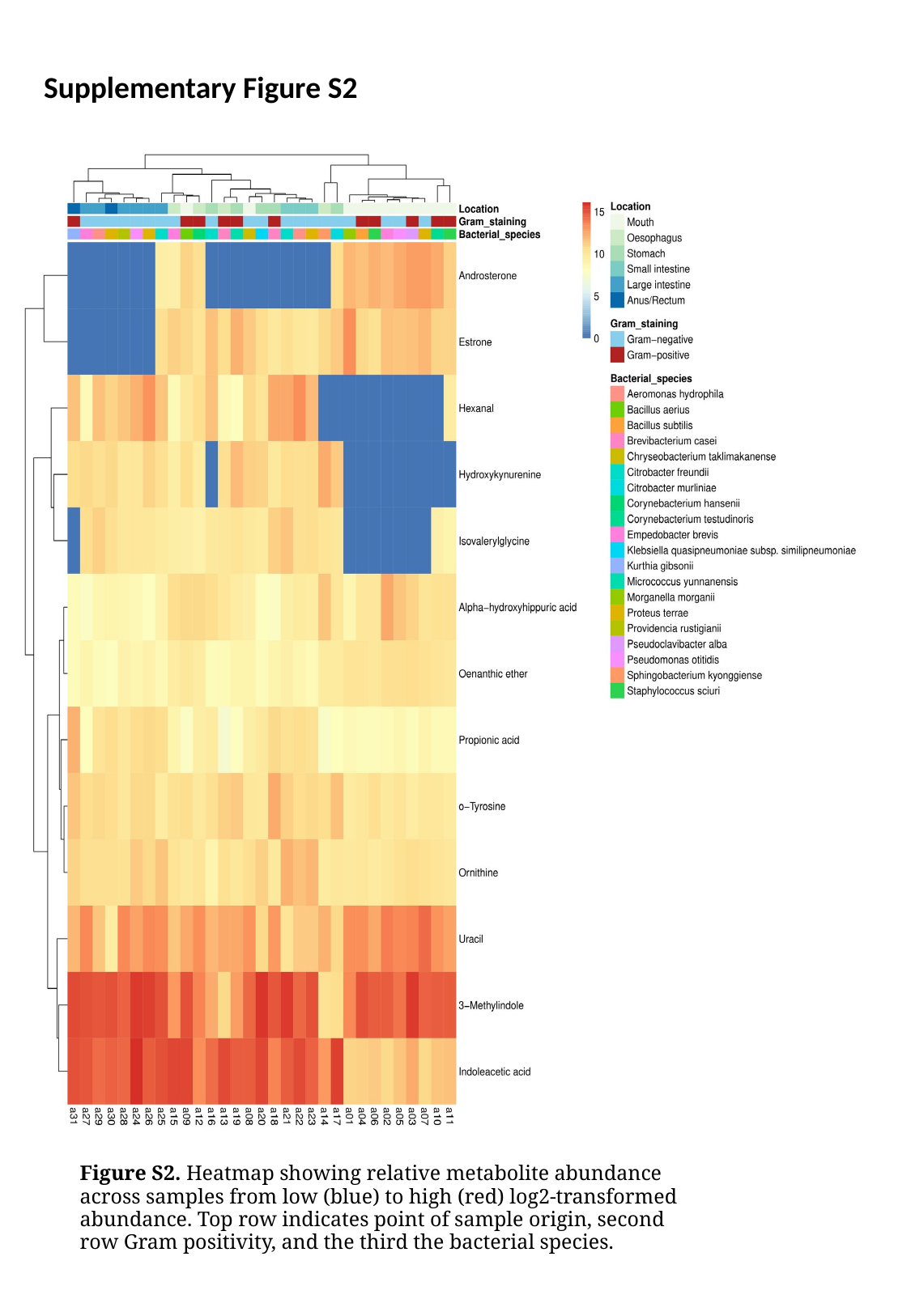

Supplementary Figure S2
Figure S2. Heatmap showing relative metabolite abundance across samples from low (blue) to high (red) log2-transformed abundance. Top row indicates point of sample origin, second row Gram positivity, and the third the bacterial species.
