# Supplementary material for: Selected Gut Bacteria from Water Monitor Lizard Exhibit Effects against Pathogenic Acanthamoeba castellanii Belonging to the T4 Genotype
Source: Microorganisms. 2023 Apr 20;11(4):1072. doi: 10.3390/microorganisms11041072 (PMC10142573; doi:10.3390/microorganisms11041072)
Supplement: Supplementary file 1 [file microorganisms-11-01072-s001.zip › Supplenetary Data_bacterial sequences.docx]

>_OQ644254: *Empedobacter brevis*

GGGGGCTACCATGCAAGCCGAGGGGTACGATTCTTTCGGGAATCTGANACCGGCGCACGGGTGCGTAACGCGTATGCAACTTGCCCTACTGAAAAGGATAGCCCTTCGAAAGGAGGATTAATACTTTATAACAGATTGAATGGCATCATTTAATTTTGAAAGATTTATCGCAGTAGGATAGGCATGCGTAAGATTAGTTAGTTGGAGAGGTAACGGCTCACCAAGACGATGATCTTTAGGGGGCCTGAGAGGGTGAACCCCCACACTGGTACTGAGACACGGACCAGACTCCTACGGGAGGCAGCAGTGAGGAATATTGGACAATGGGTGGAAGCCTGATCCAGCCATCCCGCGTGTAGGACGACTGCCTTATGGGTTGTAAACTACTTTTATCTGGGGATAAACCTACTCACGTGTGAGTAGCTGAAGGTACCAGAAGAATAAGCACCGGCTAACTCCGTGCCAGCAGCCGCGGTAATACGGAGGGTGCAAGCGTTATCCGGATTTATTGGGTTTAAAGGGTCCGTAGGCGGATTAATCAGTCAGTGGTGAAATCCCGCAGCTTAACTGTGGAACTGCCATTGATACTGTTAGTCTTGAGTGATGTTGAAGTGGCTGGAATGTGTAGTGTAGCGGTGAAATGCTTAGATATTACGCAGAACACCAATTGCGAAGGCAGGTCACTAAGTATCAACTGACGCTGATGGACGAAAGCGTGGGGAGCGAACAGGATTAGATACCCTGGTAGTCCACGCCGTAAACGATGGATACTTGCTGTTGGGGTTTCGGCTTCAGTGGCTAAGCGAAAGTTATAAGTATCCCACCTGGGGAGTACGTTCGCAAGAATGAAACTCAAAGGAATTGACGGGGGCCCGCACAAGCGGTGGAGCATGTGGTTTAATTCGATGATACGCGAGGAACCTTACCAAGGCTTAAATGCATAATGACAGATCTAGAAATAGATTTTTCTTCGGACAGAATGCAAGGTGCTGCATGGCTGTCGTCAGCTCGTGCCGTGAGGTGTTAGGTTAAGTCCTGCAACGAGCGCAACCCCTATCATTAGTTGCCAGCGTTTAAAGACGGGGACTCTAATGAGACTGCCGGTGCAAACCGCGAGGAAGGTGGGGACGACGTCAAGTCATCACGGCCCTTACGTCTTGGGCTACACACGTGCTACAATGGTAAGTACAGAGGGCAGCTACTTGGCAACAAGATGCGAATCTCNAAAACTTATCTCAGTTCGGATTGGAGTCTGCAACTCGACTCTATGAAGCTGGAATCGCTAGTAATCGCATATCAGCCATGATGCGGTGAATACGTTCCCGGGCCTTGTACACACCGCCCGTCAAGCCATGGAAGCTGGGGGTACCTGAAGTCGGTGACCGTAAAAGGAGCTGCCTAGGTAAAAC

>_OQ644256: *Pseudomonas otitidis*

CTTTAAAAAATAACNGGNGGNAGNCTANCATGCANGTCGNGCGGATGAGTGGAGCTTGCTCCATGATTCAGCGGCGGACGGGTGAGTAATGCCTAGGAATCTGCCTGGTAGTGGGGGATAACGTTTCGAAAGGAACGCTAATACCGCATACGTCCTACGGGAGAAAGTGGGGGATCTTCGGACCTCACGCTATCAGATGAGCCTAGGTCGGATTAGCTAGTTGGTGGGGTAATGGCCCACCAAGGCGACGATCCGTAACTGGTCTGAGAGGATGATCAGTCACACTGGAACTGAGACACGGTCCAGACTCCTACGGGAGGCAGCAGTGGGGAATATTGGACAATGGGCGAAAGCCTGATCCAGCCATGCCGCGTGTGTGAAGAAGGTCTTCGGATTGTAAAGCACTTTAAGTTGGGAGGAAGGGCAGTAAGTTAATACCTTGCTGTTTTGACGTTACCAACAGAATAAGCACCGGCTAACTTCGTGCCAGCAGCCGCGGTAATACGAAGGGTGCAAGCGTTAATCGGAATTACTGGGCGTAAAGCGCGCGTAGGTGGTTCAGCAAGTTGGATGTGAAAGCCCCGGGCTCAACCTGGGAATTGCATCCAAAACTACTGAGCTAGAGTACGGTAGAGGGTGGTGGAATTTCCTGTGTAGCGGTGAAATGCGTAGATATAGGAAGGAACACCAGTGGCGAAGGCGACCACCTGGACTGATACTGACACTGAGGTGCGAAAGCGTGGGGAGCAAACAGGATTAGATACCCTGGTAGTCCACGCCGTAAACGATGTCGACTAGCCGTTGGGATCCTTGAGATCTTAGTGGCGCAGCTAACGCGATAAGTCGACCGCCTGGGGAGTACGGCCGCAAGGTTAAAACTCAAATGAATTGACGGGGGCCCGCACAAGCGGTGGAGCATGTGGTTTAATTCGAAGCAACGCGAAGAACCTTACCTGGCCTTGACATGCAGAGAACTTTCCAGAGATGGATTGGTGCCTTCGGGAACTCTGACACAGGTGCTGCATGGCTGTCGTCAGCTCGTGTCGTGAGATGTTGGGTTAAGTCCCGTAACGAGCGCAACCCTTGTCCTTAGTTACCAGCACCTCGGGTGGGCACTCTAAGGAGACTGCCGGTGACAAACCGGAGGAAGGTGGGGATGACGTCAAGTCATCATGGCCCTTACGGCCAGGGCTACACACGTGCTACAATGGTCGGTACAAAGGGTTGCCAAGCCGCGAGGTGGAGCTAATCCCATAAAACCGATCGTAGTCCGGATCGCAGTCTGCAACTCGACTGCGTGAAGTCGGAATCGCTAGTAATCGTGAATCAGAATGTCNCGGTGAATACGTTCCCGGGCCTTGTACACACCGCCCGTCACACCATGGGAGTGGGTTGCTCCAGAAGTAGCTAGTCTAACCGCAAGGAGGACGGTACCCACGGAGGATT

>_OQ644257: *Pseudoclavibacter alba*

GGGGGGGGGNGNNTNNNATGCAAGTCGAACGATGAAGCCGGANCTTGCTCCGGTGGATTAGTGGCGAACGGGTGAGTAACACGTGAGCAACGTGCCCAGGACTCTGGAATAACTTCGGGAAACCGAAGCTAATACCGGATACGAGGCGAGGGGGCATCCCCATCGCCTGGAAAGAATTTCGGTTCTGGATCGGCTCACGGCCTATCAGCTTGTTGGTGAGGTAATGGCTCACCAAGGCGACGACNGGTAGCCGGCCTGAGAGGGTGACCGGCCACACTGGGACTGAGACACGGCCCAGACTCCTACGGGAGGCAGCAGTGGGGAATATTGCACAATGGGCGCAAGCCTGATGCAGCAACGCCGCGTGAGGGATGACGGCCTTCGGGTTGTAAACCTCTTTTAGTAGGGAAGAAGCGAAAGTGACGGTACCTGCAGAAAAAGCACCGGCTAACTACGTGCCAGCAGCCGCGGTAATACGTAGGGTGCAAGCGTTGTCCGGAATTATTGGGCGTAAAGAGCTCGTAGGCGGTTTGTCGCGTCTGCTGTGAAAACCCGAGGCTCAACCTCGGGCCTGCAGTGGGTACGGGCAGACTAGAGTGCAGTAGGGGAGATTGGAATTCCTGGTGTAGCGGTGGAATGCGCAGATATCAGGAGGAACACCGATGGCGAAGGCAGATCTCTGGGCTGTAACTGACGCTGAGGAGCGAAAGCATGGGGAGCGAACAGGATTAGATACCCTGGTAGTCCATGCCGTAAACGTTGGGCGCTAGATGTGGGGACCATTCCACGGTTTCCGTGTCGTAGCTAACGCATTAAGCGCCCCGCCTGGGGAGTACGGCCGCAAGGCTAAAACTCAAAGGAATTGACGGGGGCCCGCACAAGCGGCGGAGCATGCGGATTAATTCGATGCAACGCGAAGAACCTTACCAAGGCTTGACATATACCGGAAACGGCCAGAGATGGTCGCCCCGCAAGGTCGGTATACAGGTGGTGCATGGTTGTCGTCAGCTCGTGTCGTGAGATGTTGGGTTAAGTCCCGCAACGAGCGCAACCCTCGTCGTATGTTGCCAGCACGTTATGGTGGGAACTCATATGAGACTGCCGGGGTCAACTCGGAGGAAGGTGGGGATGACGTCAAATCATCATGCCCCTTATGTCTTGGGCTTCACGCATGCTACAATGGCTGGTACAAAGGGCTGCGATACCGCGAGGTGGAGCGAATCCCAAAAAGCCAGTCTCAGTTCGGATTGGGGTCTGCAACTCGACCCCATGAAGTCGGAGTCGCTAGTAATCGCAGATCAGCAACGCTGCGGTGAATACGTTCCCGGGCCTTGTACACACCGCCCGTCAAGTCATGAAAGTCGGTAACACCCGAAGCCGGTGGCCCAACCNTTGGAGGGAGCCGTCGAAGGTGGATCGANTCC

>_OQ644258: *Pseudomonas aeruginosa*

GCAGCCTACCATGCAAGTCGAGCGGATGAGTGGAGCTTGCTCCATGATTCAGCGGCGGACGGGTGAGTAATGCCTAGGAATCTGCCTGGTAGTGGGGGATAACGTTTCGAAAGGAACGCTAATACCGCATACGTCCTACGGGAGAAAGTGGGGGATCTTCGGACCTCACGCTATCAGATGAGCCTAGGTCGGATTAGCTAGTTGGTGGGGTAATGGCCCACCAAGGCGACGATCCGTAACTGGTCTGAGAGGATGATCAGTCACACTGGAACTGAGACACGGTCCAGACTCCTACGGGAGGCAGCAGTGGGGAATATTGGACAATGGGCGAAAGCCTGATCCAGCCATGCCGCGTGTGTGAAGAAGGTCTTCGGATTGTAAAGCACTTTAAGTTGGGAGGAAGGGCAGTAAGTTAATACCTTGCTGTTTTGACGTTACCAACAGAATAAGCACCGGCTAACTTCGTGCCAGCAGCCGCGGTAATACGAAGGGTGCAAGCGTTAATCGGAATTACTGGGCGTAAAGCGCGCGTAGGTGGTTCAGCAAGTTGGATGTGAAAGCCCCGGGCTCAACCTGGGAATTGCATCCAAAACTACTGAGCTAGAGTACGGTAGAGGGTGGTGGAATTTCCTGTGTAGCGGTGAAATGCGTAGATATAGGAAGGAACACCAGTGGCGAAGGCGACCACCTGGACTGATACTGACACTGAGGTGCGAAAGCGTGGGGAGCAAACAGGATTAGATACCCTGGTAGTCCACGCCGTAAACGATGTCGACTAGCCGTTGGGATCCTTGAGATCTTAGTGGCGCAGCTAACGCGATAAGTCGACCGCCTGGGGAGTACGGCCGCAAGGTTAAAACTCAAATGAATTGACGGGGGCCCGCACAAGCGGTGGAGCATGTGGTTTAATTCGAAGCAACGCGAAGAACCTTACCTGGCCTTGACATGCAGAGAACTTTCCAGAGATGGATTGGTGCCTTCGGGAACTCTGACACAGGTGCTGCATGGCTGTCGTCAGCTCGTGTCGTGAGATGTTGGGTTAAGTCCCGTAACGAGCGCAACCCTTGTCCTTAGTTACCAGCACCTCGGGTGGGCACTCTAAGGAGACTGCCGGTGACAAACCGGAGGAAGGTGGGGATGACGTCAAGTCATCATGGCCCTTACGGCCAGGGCTACACACGTGCTACAATGGTCGGTACAAAGGGTTGCCAAGCCGCGAGGTGGAGCTAATCCCATAAAACCGATCGTAGTCCGGATCGCAGTCTGCAACTCGACTGCGTGAAGTCGGAATCGCTAGTAATCGTGAATCAGAATGTCACGGTGAATACGTTCCCGGGCCTTGTACACACCGCCCGTCACACCATGGGAGTGGGTTGCNCCAGAAGTAGCTAGTCTAACCGCAAGGAGGACGGTACCACGGAGGATT

>_OQ644260: *Proteus terrae*

GGGCATGGGGCAGCCTACCATGCAAGTCGAGCGGTACAGGAGAAAGCTTGCTTTCTTGCTGACGAGCGGCGGACGGGTGAGTAATGTATGGGGATCTGCCCGATAGAGGGGGATAACTACTGGAAACGGTGGCTAATACCGCATGACGTCTACGGACCAAAGCAGGGGNTNTTNGGACCTTGCGCTATCGGATGAACCCATATGGGATTAGCTAGTAGGTGAGGTAATGGCTCACCTAGGCGACGATCTCTAGCTGGTCTGAGAGGATGATCAGCCACACTGGGACTGAGACACGGCCCAGACTCCTACGGGAGGCAGCAGTGGGGAATATTGCACAATGGGCGCAAGCCTGATGCAGCCATGCCGCGTGTATGAAGAAGGCCTTAGGGTTGTAAAGTACTTTCAGCGGGGAGGAAGGTGTTAAGATTAATACTCTTAGCATTGACGTTACCCGCAGAAGAAGCACCGGCTAACTCCGTGCCAGCAGCCGCGGTAATACGGAGGGTGCAAGCGTTAATCGGAATTACTGGGCGTAAAGCGCACGCAGGCGGTCAATTAAGTCAGATGTGAAAGCCCCGAGCTTAACTTGGGAATTGCATCTGAAACTGGTTGGCTAGAGTCTTGTAGAGGGGGGTAGAATTCCACGTGTAGCGGTGAAATGCGTAGAGATGTGGAGGAATACCGGTGGCGAAGGCGGCCCCCTGGACAAAGACTGACGCTCAGGTGCGAAAGCGTGGGGAGCAAACAGGATTAGATACCCTGGTAGTCCACGCTGTAAACGATGTCGATTTAGAGGTTGTGGTCTTGAACCGTGGCTTCTGGAGCTAACGCGTTAAATCGACCGCCTGGGGAGTACGGCCGCAAGGTTAAAACTCAAATGAATTGACGGGGGCCCGCACAAGCGGTGGAGCATGTGGTTTAATTCGATGCAACGCGAAGAACCTTACCTACTCTTGACATCCAGAGAATCCTTTAGAGATAGAGGAGTGCCTTCGGGAACTCTGAGACAGGTGCTGCATGGCTGTCGTCAGCTCGTGTTGTGAAATGTTGGGTTAAGTCCCGCAACGAGCGCAACCCTTATCCTTTGTTGCCAGCGCGTGATGGCGGGAACTCAAAGGAGACTGCCGGTGATAAACCGGAGNAAGGTGGGGATGACGTCAAGTCATCATGGCCCTTACGAGTAGGGCTACACACGTGCTACAATGGCAGATACAAAGAGAAGCGACCTCGCGAGAGCAAGCGGAACTCATAAAGTCTGTCGTAGTCCGGATTGGAGTCTGCAACTCGACTCCATGAAGTCGGAATCGCTAGTAATCGTAGATCAGAATGCTACGGTGAATACGTTCCCGGGCCTTGTACACACCGCCCGTCACACCATGGGAGTGGGTTGCAAAAGAAGTAGGTAGCTTAACCTTCGGGAGGGCGCTTACCACTTTTGATTCAG

>_OQ644261: *Chryseobacterium taklimakanense*

GGGGGGGGGANNGGGNGCTACCNTGCAAGCCGAGCGGTAGNGTTCCTTCGGGAACTTGAGAGCGGCGTACGGGTGCGGAACACGTGTGCAACCTGCCTTTATCTGGGAGATAGCCCTCCGAAAGGGGGATTAATATCCTATAATATATCGATTGGCATCAATTGATATTGAAAGCTCCGGCGGATAGAGATGGGCACGCGCAAGATTAGATAGTTGGTGAGGTAACGGCTCACCAAGTCTGCGATCTTTAGGGGGCCTGAGAGGGTGATCCCCCACACTGGTACTGAGACACGGACCAGACTCCTACGGGAGGCAGCAGTGAGGAATATTGGACAATGGGTGCAAGCCTGATCCAGCCATTCCGCGTGAAGGATTAAGGTCCTATGGATTGTAAACTTCTTTTATACAGGGATAAACCTACTTACGTGTAAGTAGCTGAAGGTACTGTATGAATAAGCACCGGCTAACTCCGTGCCAGCAGCCGCGGTAATACGGAGGGTGCAAGCGTTATCCGGATTTATTGGGTTTAAAGGGTCCGTAGGCGGGCTCGTAAGTCAGTGGTGAAAGCCTGCAGCTTAACTGTAGAACTGCCGTTGATACTGCGAGTCTTGAGTAAATTTGAAGTGGCTGGAATAAGTAGTGTAGCGGTGAAATGCATAGATATTACTTAGAACACCAATTGCGAAGGCAGGTCACTAAGATTTAACTGACGCTGATGGACGAAAGCGTGGGGAGCGAACAGGATTAGATACCCTGGTAGTCCACGCCGTAAACGATGCTAACTCGTTTTTGGGTCTTCGGGCTCAGAGACCAAGCGAAAGTGATAAGTTAGCCACCTGGGGAGTACGTTCGCAAGAATGAAACTCAAAGGAATTGACGGGGGCCCGCACAAGCGGTGGATTATGTGGTTTAATTCGATGATACGCGAGGAACCTTACCAAGACTTAAATGGGAGTGGACAGGCTTAGAAATAGGCTCTCCTTCGGGCCGCTTCCAAGGTGCTGCATGGTTGTCGTCAGCTCGTGCCGTGAGGTGTTAGGTTAAGTCCTGCAACGAGCGCAACCCCTGTCACTAGTTGCTAACATTCAGTTGAGGACTCTAGTGAGACTGCCTACGCNAGTAGAGAGGAAGGTGGGGATGACGTCAAATCATCACGGCCCTTACGTCTTGGGCCACACACGTAATACAATGGCCGGTACAGAGGGCAGCTACACAGCGATGTGATGCNAATCTCGAAAGCCGGTCTCAGTTCGGATTGGAGTCTGCAACTCGACTCTATGAAGCTGGAATCGCTAGTAATCGCGCATCAGCCATGGCGCGGTGAATACGTTCCCGGGCCTTGTACACACCGCCCGTCAAGCCATGGAAGTNTGGGGTACCTGAAGTCGGTGACCGTAAAAGGAGCTGCCTAGGNAAANAAAGGAGTTTTTTTTTAAAAAAAAC

>_OQ644262: *Bacillus aerius*

CGGCNNCCTATACATGCAAGTCGAGCGGACAGAAGGGAGCTTGCTCCCGGATGTTAGCGGCGGACGGGTGAGTAACACGTGGGTAACCTGCCTGTAAGACTGGGATAACTCCGGGAAACCGGAGCTAATACCGGATAGTTCCTTGAACCGCATGGTTCAAGGATGAAAGACGGTTTCGGCTGTCACTTACAGATGGACCCGCGGCGCATTAGCTAGTTGGTGAGGTAACGGCTCACCAAGGCGACGATGCGTAGCCGACCTGAGAGGGTGATCGGCCACACTGGGACTGAGACACGGCCCAGACTCCTACGGGAGGCAGCAGTAGGGAATCTTCCGCAATGGACGAAAGTCTGACGGAGCAACGCCGCGTGAGTGATGAAGGTTTTCGGATCGTAAAGCTCTGTTGTTAGGGAAGAACAAGTGCAAGAGTAACTGCTTGCACCTTGACGGTACCTAACCAGAAAGCCACGGCTAACTACGTGCCAGCAGCCGCGGTAATACGTAGGTGGCAAGCGTTGTCCGGAATTATTGGGCGTAAAGGGCTCGCAGGCGGTTTCTTAAGTCTGATGTGAAAGCCCCCGGCTCAACCGGGGAGGGTCATTGGAAACTGGGAAACTTGAGTGCAGAAGAGGAGAGTGGAATTCCACGTGTAGCGGTGAAATGCGTAGAGATGTGGAGGAACACCAGTGGCGAAGGCGACTCTCTGGTCTGTAACTGACGCTGAGGAGCGAAAGCGTGGGGAGCGAACAGGATTAGATACCCTGGTAGTCCACGCCGTAAACGATGAGTGCTAAGTGTTAGGGGGTTTCCGCCCCTTAGTGCTGCAGCTAACGCATTAAGCACTCCGCCTGGGGAGTACGGTCGCAAGACTGAAACTCAAAGGAATTGACGGGGGCCCGCACAAGCGGTGGAGCATGTGGTTTAATTCGAAGCAACGCGAAGAACCTTACCAGGTCTTGACATCCTCTGACAACCCTAGAGATAGGGCTTTCCCTTCGGGGACAGAGTGACAGGTGGTGCATGGTTGTCGTCAGCTCGTGTCGTGAGATGTTGGGTTAAGTCCCGCAACGAGCGCAACCCTTGATCTTAGTTGCCAGCATTCAGTTGGGCACTCTAAGGTGACTGCCGGTGACAAACCGGAGGAAGGTGGGGATGACGTCNAATCATCATGCCCCTTATGACCTGGGCTACACACGTGCTACAATGGACAGAACAAAGGGCTGCGAGACCGCAAGGTTTAGCCAATCCCACAAATCTGTTCTCAGTTCGGATCGCAGTCTGCAACTCGACTGCGTGAAGCTGGAATCGCTAGTAATCGCGGATCAGCATGCCGCGGTGAATACGTTCCCGGGCCTTGTACACACCGCCCGTCACACCACGAGAGTTTGCANCACCCGAAGTCGGTGAGGTAACCNTTNTGGAGCCAGCCNCCGAAGGNGGGCAGA

>_OQ644263: *Morganella morganii*

GGGCNGGCGGCAGGCCTACCATGCAAGTCGGGCGGTACAGGGAAGAAGCTTGCTTCTCTGCTGACGAGCGGCGGACGGGTGAGTAATGTATGGGGATCTGCCTGATGGCGGGGGATAACTACTGGAAACGGTAGCTAATACCGCATAATGTCTTCGGACCAAAGCGGGGGACCTCCGGGCCTCGCGCCATCAGATGAACCCATATGGGATTAGCTAGTAGGTGAGGTAACGGCTTACCTAGGCGACGATCCCTAGCTGGTCTGAGAGGATGATCAGCCACACTGGGACTGAGACACGGCCCAGACTCCTACGGGAGGCAGCAGTGGGGAATATTGCACAATGGGCGCAAGCCTGATGCAGCCATGCCGCGTGTATGAAGAAGGCCTTCGGGTTGTAAAGTACTTTCAGTCGGGAGGAAGGTGTCAAGGTTAATAACCTTGGCAATTGACGTTACCGACAGAAGAAGCACCGGCTAACTCCGTGCCAGCAGCCGCGGTAATACGGAGGGTGCAAGCGTTAATCGGAATTACTGGGCGTAAAGCGCACGCAGGCGGTTGATTGAGTCAGATGTGAAATCCCCGGGCTTAACCCGGGAATTGCATCTGATACTGGTCAGCTAGAGTCTTGTAGAGGGGGGTAGAATTCCATGTGTAGCGGTGAAATGCGTAGAGATGTGGAGGAATACCGGTGGCGAAGGCGGCCCCCTGGACAAAGACTGACGCTCAGGTGCGAAAGCGTGGGGAGCAAACAGGATTAGATACCCTGGTAGTCCACGCTGTAAACGATGTCGACTTGGAGGTTGTGCCCTTGAGGCGTGGCTTCCGGAGCTAACGCGTTAAGTCGACCGCCTGGGGAGTACGGCCGCAAGGTTAAAACTCAAATGAATTGACGGGGGCCCGCACAAGCGGTGGAGCATGTGGTTTAATTCGATGCAACGCGAAGAACCTTACCTACTCTTGACATCCAGAGAACTTAGCAGAGATGCTTTGGTGCCTTCGGGAACTCTGAGACAGGTGCTGCATGGCTGTCGTCAGCTCGTGTTGTGAAATGTTGGGTTAAGTCCCGCAACGAGCGCAACCCTTATCCTTTGTTGCCAGCGCGTGATGGCGGGAACTCAAAGGAGACTGCCGGTGATAAACCGGAGGAAGGTGGGGATGACGTCAAGTCATCATGGCCCTTACGAGTAGGGCTACACACGTGCTACAATGGCGTATACAAAGGGAAGCGACCCCGCGAGGGCAAGCGGAACTCATAAAGTACGTCGTAGTCCGGATTGGAGTCTGCAACTCGACTCCATGAAGTCGGAATCGCTAGTAATCGTAGATCAGAATGCTACGGTGAATACGTTCCCGGGCCTTGTACACACCGCCCGTCACACCATGGGAGTGGGTTGCAAAAGAAGTAGGTAGCTTAACCTCCGGGAGGGCGCTTACCACTTGATC

>_OQ644264: *Corynebacterium atrinae*

GGGCNGCTTACCATGCAAGTCGAACGGAAANGCCTNCNAGCTNNGNCTGGGGTACTCNAGTGGNGAACGGGNGANNAACACGTGGGTNATCTGCCTCGNACTTCNNGATAAGCTTNGGAAACTGGGTCTAATACCGGATATAACCNGCCTTTAGTGTGGTGGGTGGAAAGTTTTGTCGGTACGAGNTGAGCCCGCGGCCTATCAGCTTGTTGGTGGGGTNATGGCCTACCNAGGNGTCGACNGGTAGCCNGCCTGAGAGGGTNGACGNCCNCANTGGGANTGAGATACGGCCCNGACTCCTACNGGAGGCAGCAGTNGGGAATATTGCACNATGGGNGNCAANCCTGATGCAGNGACGCCGCGTNGGGGATGACGGCCTTCGGGTTGTAAACCTCTTTCGCTAGGGACGAAGCTGNCCTTTTGGGGTGGTGACGGTACCTGGATAAGAAGCACCGGCTAACTACNTGCCAGCAGCCGCGGTAATACGTAGGGTGCGAGCGTTGTCCGGAATTACTGGNCGTAAAGAGCTCGTAGGTGGTTTGTCGCGTCGTCTGTGAAATTCCGGGGCTTAACTCCGGGCGTGCAGGCGATACGGGCAATACTTGAGTGCTGTAGGGGAGACTGGAATTCCTGGTGTAGCGGTGAAATGCGCAGATATCAGGAGGAACACCAATGGCGAAGGCAGGTCTCTGGGCAGTAACTGACGCTGAGGAGCGAAAGCATGGGTAGCGAACAGGATTAGATACCCTGGTAGTCCATGCCGTAAACGGTGGGCGCTAGGTGTAGGGGTCTTCCACGACTTCTGTGCCGTAGCTAACGCATTAAGCGCCCCGCCTGGGGAGTACGGCCGCAAGGCTAAAACTCNAAGGAATTGACGGGGGCCCGCACAAGCGGCGGAGCATGTGGATTAATTCGATGCAACGCGAAGAACCTTACCTGGGCTTGACATATACGGGATCGCCGCAGAGATGTGGTTTCCCTTGTGGCTCGTATACAGGTGGTGCATGGTTGTCGTCAGCTCGTGTCNTGAGATGTTGGGTTAAGTCCCGCAACGAGCGCAACCCTTGTCTTATGTTGCCAGCACGNTANAGGTGGGGACTCATGANAGACTGCCGGGGTCAACTCGGAGGAAGGNGGGGATGNACGTCAAATCATCATGCCCCTTATGTCCAGGGCTTNCACACATGCTACAATGGTCGGTACAACGCGTTGCCNGCCCNTGAGGGTGCGCTAATCNCNTGAAAGCCGGCCTCANNTCGNANTGGGGTCTGCAACTCGACCCCATGAANTCGGAGTCGCTAGNAATCNCAGATCAGNCANCGCTGCGGTGAATACGTTCCNGGGCNTTGTACACNCCGCCCGNCACGTCNTGAAAGTTGGTAACNCCCGAAGCCAGTGGCCCAACCCTATGTGAGGGAGCTGTCGCAAGGTNATCCGCAGGGC

>_OQ644265: *Mammaliicoccus sciuri*

CCTATACATGCAAGTCGAGCGAACAGATGAGAAGCTTGCTTCTCTNATGTTAGCGGCGGNNGGGTGAGTAACACGTGGGTAACCTACCTATAAGACTGGGATAACTCCGGGAAACCGGGGCTAATACCGGATAATATTTTGAACCGCATGGTTCAATAGTGAAAGACGGTTTCGGCTGTCACTTATAGATGGACCCGCGCCGTATTAGCTAGTTGGTAAGGTAANGGCTTACCAAGGCGACGATACGTAGCCGACCTGAGAGGGTGATCGGCCACACTGGAACTGAGACACGGTCCAGACTCCTACGGGAGGCAGCAGTAGGGAATCTTCCGCAATGGGCGAAAGCCTGACGGAGCAACGCCGCGTGAGTGATGAAGGTCTTCGGATCGTAAAACTCTGTTGTTAGGGAAGAACAAATTTGTTAGTAACTGAACAAGTCTTGACGGTACCTAACCAGAAAGCCACGGCTAACTACGTGCCAGCAGCCGCGGTAATACGTAGGTGGCAAGCGTTATCCGGAATTATTGGGCGTAAAGCGCGCGTAGGCGGTTTCTTAAGTCTGATGTGAAAGCCCACGGCTCAACCGTGGAGGGTCATTGGAAACTGGGAAACTTGAGTGCAGAAGAGGAGAGTGGAATTCCATGTGTAGCGGTGAAATGCGCAGAGATATGGAGGAACACCAGTGGCGAAGGCGGCTCTCTGGTCTGTAACTGACGCTGATGTGCGAAAGCGTGGGGATCAAACAGGATTAGATACCCTGGTAGTCCACGCCGTAAACGATGAGTGCTAAGTGTTAGGGGGTTTCCGCCCCTTAGTGCTGCAGCTAACGCATTAAGCACTCCGCCTGGGGAGTACGACCGCAAGGTTGAAACTCAAAGGAATTGACGGGGACCCGCACAAGCGGTGGAGCATGTGGTTTAATTCGAAGCAACGCGAAGAACCTTACCAAATCTTGACATCCTTTGACCGCTCTAGAGATAGAGTCTTCCCCTTCGGGGGACAAAGTGACAGGTGGTGCATGGTTGTCGTCAGCTCGTGTCGTGAGATGTTGGGTTAAGTCCCGCAACGAGCGCAACCCTTAAGCTTAGTTGCCATCATTAAGTTGGGCACTCTAGGTTGACTGCCGGTGACAAACCGGAGGAAGGTGGGGATGACGTCNAATCATCATGCCCCTTATGATTTGGGCTACACACGTGCTACAATGGATAATACAAAGGGCAGCGAATCCGCGAGGCCAAGCAAATCCCATAAAATTATTCTCAGTTCGGATTGTAGTCTGCAACTCGACTACATGAAGCTGGAATCGCTAGTAATCGTAGATCAGCATGCTACGGTGAATACGTTCCCGGGTCTTGTACACACCGCCCGTCACACCACGAGAGTTTGTAACNCCCGAAGCCGGTGGAGTAACCTTTAGGAGCNNNCCNNCNAAGGNNGACANTGGGGGGGGTTTTTTTAAAAAAAAAAAAAA

>_OQ644266: *Chryseobacterium taklimakanense*

GGGAGCTACCATGCAAGCCGAGCGGTAGAGCTCCTTCGGGATCTTGAGAGCGGCGTACGGGTGCGGAACACGTGTGCAACCTGCCTTTATCTGGGAGATAGCCCACCGAAAGGTGGATTAATATTCCATAATATATTGATTGGCATCGATTAATATTGAAAGCTCCGGCGGATAGAGATGGGCACGCGCAAGATTAGATAGTTGGTGAGGTAACGGCTCACCAAGTCTGCGATCTTTAGGGGGCCTGAGAGGGTGATCCCCCACACTGGTACTGAGACACGGACCAGACTCCTACGGGAGGCAGCAGTGAGGAATATTGGACAATGGGTGCAAGCCTGATCCAGCCATCCCGCGTGAAGGATTAAGGTCCTATGGATTGTAAACTTCTTTTATACAGGGATAAACCTACTTACGTGTAAGTAGCTGAAGGTACTGTATGAATAAGCACCGGCTAACTCCGTGCCAGCAGCCGCGGTAATACGGAGGGTGCAAGCGTTATCCGGATTTATTGGGTTTAAAGGGTCCGTAGGCGGGCTCGTAAGTCAGTGGTGAAAGCCTGCAGCTTAACTGTAGAACTGCCGTTGATACTGCGAGTCTTGAGTAAATTTGAAGTGGCTGGAATAAGTAGTGTAGCGGTGAAATGCATAGATATTACTTAGAACACCAATTGCGAAGGCAGGTCACTAAAATTTAACTGACGCTGATGGACGAAAGCGTGGGGAGCGAACAGGATTAGATACCCTGGTAGTCCACGCCGTAAACGATGCTAACTCGTTTTTGGGCACTTGTGTTCAGAGACCAAGCGAAAGTGATAAGTTAGCCACCTGGGGAGTACGTTCGCAAGAATGAAACTCAAAGGAATTGACGGGGGCCCGCACAAGCGGTGGATTATGTGGTTTATTCGATGATACGCGAGGAACCTTACCAAGACTTAAATGGGAGTGGACAGGCTTAGAAATAGGTCTTCCTTCGGGTCGCTTCCAAGGTGCTGCATGGTTGTCGTCAGCTCGTGCCGTGAGGTGTTAGGTTAAGTCCTGCAACGAGCGCAACCCCTGTCACTAGTTGCTAACATTTAGTTGAGGACTCTAGTGAGACTGCCTACGCNAGTAGAGAGGAAGGTGGGGATGACGTCAAATCATCACGGCCCTTACGTCTTGGGCCACACACGTAATACAATGGCCGGTACAGAGGGCAGCTACACAGCGATGTGATGCAAATCTCGAAAGCCGGTCTCAGTTCGGATTGGAGTCTGCAACTCGACTCTATGAAGCTGGAATCGCTAGTAATCGCGCATCAGCCATGGCGCGGTGAATACGTTCCCGGGCCTTGTACACNCCGCCCGTCAAGCCATGGAAGTCTGGGGTACCTGAAGTCGGTGACCGTAAAAGGAGCTGCCTAGGTAAAA

>_OQ644267: *Corynebacterium freneyi*

GGCGNCTTAACCATGCAAGTCGAACGGTAAGGCCCCAGCTTGCTGGGGTACACGAGTGGCGAACGGGTGAGTAACACGTGGGTGACCTGCCCCGCACTTCGGGATAAGCCTGGGAAACTGGGTCTAATACCGGATAGGACCGCACCGTGAGGGTGTGGTGGAAAGTTTTTCGGTGTGGGATGGGCCCGCGGCCTATCAGCTTGTTGGTNGGGTAATGGCCTACCAAGGCGGCGACGGGTAGCCGGCCTGAGAGGGTGGACGGCCACATTGGGACTGAGACACGGCCCAGACTCCTACGGGAGGCAGCAGTNGGGAATATTGCACAATGGGCGGAAGCCTGATGCAGCGACGCCGCGTGGGGGATGACGGCCTTCGGGTTGTAAACTCCTTTCACCATCGACGAAGGTTTTCTGACGGTAGATGGAGAAGAAGCACCGGCTAACTACGTGCCAGCAGCCGCGGTAATACGTAGGGTGCGAGCGTTGTCCGGAATTACTGGGCGTAAAGAGCTCGTAGGTGGTTTGTCGCGTCGTCTGTGAAATTCCGGGGCTTAACTCCGGGCGTGCAGGCGATACGGGCATAACTTGAGTACTGTAGGGGAGACTGGAATTCCTGGTGTAGCGGTGAAATGCGCAGATATCAGGAGGAACACCGGTGGCGAAGGCGGGTCTCTGGGCAGTAACTGACGCTGAGGAGCGAAAGCATGGGTAGCGAACAGGATTAGATACCCTGGTAGTCCATGCCGTAAACGGTGGGCGCTAGGTGTAGGGGTCTTCCACGACTTCTGTGCCGTAGCTAACGCATTAAGCGCCCCGCCTGGGGAGTACGGCCGCAAGGCTAAAACTCAAAGGAATTGACGGGGGCCCGCACAAGCGGCGGAGCATGTGGATTAATTCGATGCAACGCGAAGAACCTTACCTGGGCTTGACATGTACGGGACCGCCGCAGAGATGTGGTTTCCCTTGTGGCTCGTATACAGGTGGTGCATGGTTGTCGTCAGCTCGTGTCGTGAGATGTTGGGTTAAGTCCCGCAACGAGCGCAACCCTTGTCTTATGTTGCCAGCACGTAATGGTGGGGACTCGTGAGAAACTGCCGGGGTCAACTCGGAGGAAGGTGGGGATGACGTCAAATCATCATGCCCCTTATGTCCAGGGCTTCACACATGCTACAATGGTCGGTACAGTGGGTTGCGATACCGTGAGGTGGAGCTAATCCCTTAAAGCCGGTCTCAGTTCGGATCGGGGTCTGCAACTCGACCCCGNGAAGTCGGAGTCGCTAGTAATCGCAGATCAGCAACGCTGCGGTGAATACGTTNCCGGGCCTTGTACACNCCGCCCGTCACGTCATGAAAGTCGGTAACACCCGAAGCCNGTGGCCCAACCTTTTAGGGGGGAGCTGTCGAAGGTGGATCGCA
